# Fractal-like gold nanonetworks formed by templated electrodeposition through 3D-mesoporous silica films†

**DOI:** 10.1039/d3ra06588j

**Published:** 2023-11-06

**Authors:** Li Shao, Gilles E. Moehl, Ruomeng Huang, Andrew L. Hector

**Affiliations:** a School of Chemistry, University of Southampton, Highfield Southampton SO17 1BJ UK a.l.hector@soton.ac.uk li.shao@soton.ac.uk; b School of Electronics and Computer Science, University of Southampton Southampton SO17 1BJ UK

## Abstract

Fractal-like networks of gold nanoparticles created by templated electrodeposition are described. Templated electrodeposition is a powerful and efficient technique for the bottom-up fabrication of nanostructures which can effectively control the size and shape of the electrodeposits. In this work, mesoporous silica films with highly ordered mesopores and three-dimensional mesostructure are synthesised and are used as templates for the electrodeposition of gold nanoparticles. The mesoporous silica films have small mesopores (∼8 nm) and complex mesopore channels (*Fmmm* structure with the [0 1 0] axis perpendicular to the substrate). A variety of nucleation conditions were applied to investigate their effect on the nanoparticles' arrangement and growth in templated electrodeposition. The electrodeposited gold particles are characterised by electron microscopy and grazing incidence small-angle X-ray scattering (GISAXS). GISAXS shows changes in the lattice parameters of the mesostructure after gold electrodeposition that relate to dimensional changes in directions linked to the shortest distances between the main spherical pores. Top-view SEM shows large areas of gold nanoparticles were deposited into the film and they were growing towards the surface. After removing the silica film templates, the gold nanoparticles display interesting fractal morphologies: the linked gold nanonetworks form a branched structure. The lengths of branches vary from the applied nucleation deposition conditions. Generally, with increasing nucleation time, fractal gold nanoparticles with longer branches are more likely to be obtained.

## Abstract 2

Fractal-like networks of gold nanoparticles created by templated electrodeposition are described.

## Graphical abstract

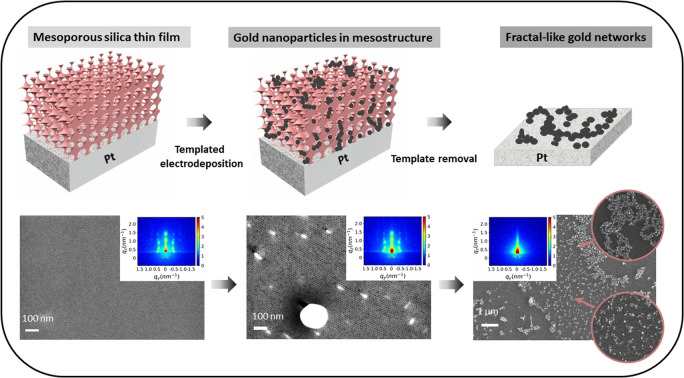

## Introduction

Gold nanoparticles have attracted considerable scientific and technological attention due to their ease of synthesis, high chemical and physical stability, surface plasmon resonance and low toxicity.^1,2^ They have been widely applied in biotechnology,^3^ catalysis^4–6^ and optoelectronics.^7^ The properties of gold nanoparticles can be strongly altered by changing the nanoparticle sizes, shapes and aspect ratios.^8,9^ Fractal-like gold nanoparticles have high surface areas, special shapes and unique physical and chemical properties, making them attract great interest in a variety of fields such as surface-enhanced Raman scattering,^10^ biomedical,^11^ stretchable transparent electronics,^12^ detection of traces of molecules,^13^ catalysis,^14^ and so on. Various methods have been developed to fabricate fractal gold structures, such as hydrothermal reduction,^15^ seed-mediated growth,^16^ and electrodeposition.^14^

Electrodeposition is able to fabricate nanostructured materials with morphologies, sizes and the compositions tuned by controlling the deposition parameters such as current density, applied potential (constant or pulsed) and duration. Specifically, templated electrodeposition provides a versatile method to fabricate well-defined nanostructures.^17,18^ Commercial track-etched polycarbonate membranes and porous anodic aluminium oxide membranes with vertical pore channels and >20 nm pore sizes are the most commonly used templates for electrodeposition. Silica thin films have excellent insulation and thermal stability properties, and are widely used as a dielectric material.^19^ Mesoporous silica possesses short channels of highly accessible active sites.^20–23^ Therefore, mesoporous silica films can be excellent candidates as templates benefiting from their insulation, tuneable pore sizes and pore structures. By tailoring the surfactants for synthesis of mesoporous silica films, mesopore sizes of 2 to 30 nm with different mesostructures can be obtained. However, reports of fabrication of metal and polymer nanostructures through electrodeposition with mesoporous silica films templates are limited.^18,24–27^

The early stages of electrochemical nucleation and growth have been studied for decades.^28–30^ A nucleus consists of a cluster of atoms, it is only stable when it exceeds a critical size. The growth of individual nuclei is determined by the rate of addition of new atoms and overlaps in growth centres.^28,31,32^ A few models of the current transients obtained with three-dimensional electrochemical nucleation on a finite number of active sites have been proposed and refined, including by Scharifker–Hills,^33^ Scharifker–Mostany,^34^ Mirkin and Nilov^35^ and Heerman and Tarallo.^28^ These models show the depositing metal nuclei are dispersed narrowly at first then the size dispersion increases as they increase in size. Liu *et al.* proposed a general method to electrodeposit metal nano- and micro-particles with narrow disparity by applying a short but more negative potential to nucleate metal particles.^36^ This method is very useful for templated electrodeposition. Applying a short and more negative pulse can create more nuclei at the bottom of a template, then growth pulses with less negative potential allow the nuclei to grow slowly along the pores. This can avoid aggregation of the gold particles and destruction of the templates and so can obtain the gold nanoparticles with better uniformity. Macpherson *et al.* investigated the initial stages of gold nucleation during electrodeposition from the deposition of an individual gold atom to formation of a crystalline nanoparticle. They observed potential-induced atom movement, atom clustering and the formation of crystalline gold nanoparticles through identical location scanning transmission electron microscopy (IL-STEM) combined with boron doped diamond (BDD) TEM electrodes.^37^ Very recently, Laskowski *et al.* produced ultra-thin silver nanowires (no more than 2 nm in diameter) in vertically aligned mesoporous silica films by fabricating organically-functionalized silica thin films with silver ions distributed in the mesopores, followed by thermal decomposition.^38^

Grazing incidence small-angle X-ray scattering (GISAXS) is a technique to study nanostructured surfaces and thin films.^39^ Compared to other structure measuring techniques such as AFM or SEM, which generally only measure the film surface, GISAXS provides information on ordered structures lateral and normal to the substrate and inside the thin films rather than just at their surface. GISAXS is a powerful technique to investigate the arrangement of electrodeposited gold nanoparticles in mesoporous thin films. A few studies investigated the nucleation and growth of gold nanoparticles fabricated by chemical reduction method through small-angle X-ray scattering.^40^ Moehl *et al.* have explored the spatial arrangement of Au particles generated through aqueous electrodeposition on a non-templated substrate by GISAXS, showing that the spatial arrangement of electrodeposited gold nanoparticles has a non-random distribution.^41^

In this work, fractal-like gold nanonetworks of gold nanoparticles were fabricated by templated electrodeposition and the effect of nucleation process on the arrangement of the deposited gold within a three-dimensional mesostructured silica film template was explored by using GISAXS and SEM. Firstly, mesoporous silica thin films with *Fmmm* mesostructure were fabricated on TiN substrates through the evaporation-induced self-assembly method. Two-step pulsed electrodeposition was applied to deposit gold nanoparticles on TiN substrates through mesoporous silica films. First, a short negative pulse was applied to generate nuclei, followed by application of pulses with less negative potential to grow the particles. GISAXS was conducted to study the arrangement of gold particles in and on the thin films. To then observe the gold particles more directly, the mesoporous silica films were removed by HF vapour etching. SEM was used to observe the morphologies and arrangements of the electrodeposited gold nanoparticles.

## Experimental

98% tetraethoxysilane (TEOS) was obtained from Alfa Aesar. Triblock copolymer F127, sodium hydroxide, potassium chloride, potassium tetrachloroaurate (iii), hydrochloric acid (37 wt% in water) and hydrofluoric acid (48 wt% in water) were bought from Sigma-Aldrich. Solvents isopropanol (99.5%), dichloromethane (99%) and absolute ethanol (99.8%) were from Fisher Scientific. Deionised water (18 MΩ cm) was from a suez select fusion machine. Titanium nitride (TiN) substrates were made by sputtering 200 nm of TiN onto a 700 mm thick silicon wafer, the substrate size is 8 × 20 mm.

The silica deposition sol was prepared using a method developed by Zhao *et al.*^42^ 1.0 g TEOS was dissolved into 5.64 g ethanol, 0.80 g deionised water and 0.10 g of 1 mol dm^−3^ hydrochloric acid at 338 K and stirred for 45 min. 0.3024 g triblock copolymer F127 was dissolved into 5.64 g ethanol. The precursor solution was prepared by mixing the above two solutions and stirring at room temperature for 60 min. TiN substrates were cleaned with isopropanol and deionized water by ultrasonication and were dried with a nitrogen jet. Dip-coating was used to coat the silica films onto the TiN substrates. The cleaned TiN substrates were vertically immersed into the prepared precursor solution and withdrawn at 150 mm min^−1^ under 75% relative humidity at 298 K. The as-made films were aged at 120 °C for 10 h. The surfactant F127 was removed by bathing the films in dichloromethane for 4 h and calcining in air at 350 °C for 5 h (temperature ramp was 1 °C min^−1^).

Before being used as the templates for electrodeposition, TiN substrates coated with mesoporous silica films were dried at 120 °C under vacuum overnight to remove the moisture in the pores. The electrolyte solution was 0.5 mol dm^−3^ K[AuCl_4_] and 100 mol dm^−3^ KCl in 50 mL deionised water. The electrochemical techniques were performed with a three-electrode electrochemical cell and a Biologic SP-150 potentiostat controlled with ECLab software. A bare TiN substrate or a mesoporous silica film on a TiN substrate was the working electrode, with a Pt mesh counter electrode and a home-made Ag/AgCl reference electrode (saturated KCl in deionised water). The reaction area of the working electrode was confined to 12.56 mm^2^ with an O-ring. Two-step pulsed electrodepositions were carried out to grow gold into the mesoporous silica films, firstly applying nucleation potentials of −1.0 V, −1.5 V or −1.8 V for 0.1 s or 1.0 s, followed by growth at −0.1 V for 1 s for 100 cycles. The pulse off time was 10 seconds. The same electrodeposition conditions were applied on the bare TiN substrates for comparison. After characterisation, mesoporous silica films were etched away by exposing to 48% hydrofluoric acid vapour for 20 min.

GISAXS and XRD experiments were conducted with a Rigaku Smartlab using a Hypix-3000 Detector System and Cu-K_α_ radiation (*λ* = 1.54 Å, 8 keV). The distance between the sample and the detector surface was around 300 mm (measured for every experiment). For GISAXS, the incident angle was 0.3° for the mesoporous silica film, slightly above the critical angle of silica (0.224°) and was 0.2° for the gold samples after removal of the silica films.^43^ The exposure time for each test was 20 min. The SEM images were collected with a ZEISS Sigma 500 VP FE-SEM except for Fig. S6,† which were collected with a JEOL JSM-6500F microscope. Energy-dispersive X-ray (EDX) mapping and spectra were collected with a ZEISS Sigma 500 VP FE-SEM.

## Results and discussion

### Electrochemistry

**Fig. 1:**
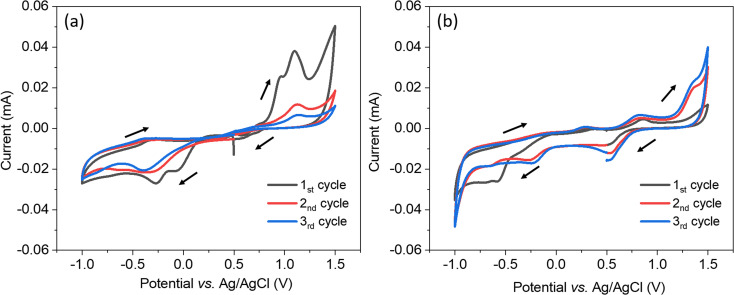
Cyclic voltammograms obtained from an electrolyte of 0.5 mol dm^−3^ K[AuCl_4_] and 100 mol dm^−3^ KCl aqueous solution on a bare TiN substrate (a) and on a TiN substrate coated with a mesoporous silica film (b). The scan rate was 50 mV s^−1^. The reactive area was controlled as 12.56 mm^2^.

The electrochemical system was characterised using cyclic voltammetry scanning from 0.5 V to −1.0 V *vs.* Ag/AgCl, then to 1.5 V and back to 0.5 V with a scan rate of 50 mV s^−1^. In Fig. 1(a), when using the bare TiN as the working electrode, the voltammetry started with two reduction peaks at 0.07 and −0.27 V indicating there were two reduction reactions of [AuCl_4_]^−^, they are likely from Au(iii) reducing to Au(i) then reducing to Au(0).^41^ In the second and third scans, there was only one reduction peak which shifted to more negative potentials. The reduction peak is likely from Au(iii) reducing to Au(0).^41^ A shift to smaller overpotential is often observed as deposition progresses since it is thermodynamically easier to deposit gold onto a gold surface than onto TiN surface, so the stripping of gold in the oxidation step seems to be removing everything. The more difficult deposition in the second and third scan was probably because of a less conductive surface due to the partial oxidisation of TiN in the high potential parts of the CV. Therefore, positive overpotentials are avoided in the electrodeposition. In Fig. 1(b), when using the mesoporous silica film dip-coated on TiN as the working electrode, the voltammetry started with a gradually increasing negative current and the reduction peak appeared at −0.57 V. The reduction peaks were more negative than the peaks seen with the bare TiN substrate because of the diffusion limitation in the mesostructure. In the first scan, the reduction peak area is bigger than the oxidation peak area, revealing that not all of the gold was stripped. In the second and third scan, several reduction peaks were observed at 0.51 V, −0.26 V and −0.66 V *vs.* Ag/AgCl, they shifted to more positive potentials than the first scan (smaller overpotential) because gold particles were electrodeposited on gold. The deposition current using the TiN substrate covered with silica film was only slightly lower than for the bare TiN substrate, demonstrating that most of the mesopores are accessible and the mesoporous silica film is suitable to be an electrodeposition template.

To carry out a uniform templated electrodeposition, nuclei need to form at the bottom of as many of the pore channels in the mesoporous silica film template as possible, and then grow simultaneously to avoid faster deposition in regions where deposits have already grown closer to the template/electrolyte interface. Thus, it is necessary to avoid growth under diffusion control. In this work, a two-step pulsed electrodeposition process was applied to maximise the likelihood of the gold growing through the mesopores slowly and uniformly. A short potential pulse at a more negative potential was firstly applied for the creation of nuclei. After one (the first) pulse-off time, growth pulses with a less negative potential were applied to allow the nuclei to grow smoothly. Between each pulse-on time of growth electrodeposition, a long pulse-off time (10 s) was used to allow the concentration of the precursor species in the pores to return to a homogeneous state, in this way diffusion control can be avoided and a high deposition rate can be obtained during each on-time pulse. Previously we described how the nucleation process can affect the arrangement of deposits.^41^ In this work, the main variation examined was in the nucleation process: gold electrodeposition was conducted by applying nucleation potentials at −1.0 V, −1.5 V or −1.8 V for 0.1 s or 1.0 s, followed by growth at −0.1 V for 1.0 s (pulse-on time) then at open circuit potential for 10.0 s (pulse-off time) for 100 repeated cycles. The duty circle is 10%. The electrodeposition details of each sample are listed in Table 1. The current transient of one sample (nucleation at −1.8 V for 1.0 s) is shown in Fig. S1† as a typical example.

**Table 1:** The applied conditions of pulsed electrodeposition on mesoporous silica films and the transferred charge calculated from *I* × *t*

Sample no.	Nucleation process		Growth process			*Q* (mC)
	Potential/V	Duration/s	Potential/V	Duration/s	Repeated cycle times	
1	−1.0	0.1	−0.1	1.0	100	−9.04
2	−1.0	1.0	−0.1	1.0	100	−15.80
3	−1.5	0.1	−0.1	1.0	100	−10.76
4	−1.5	1.0	−0.1	1.0	100	−12.94
5	−1.8	0.1	−0.1	1.0	100	−13.90
6	−1.8	1.0	−0.1	1.0	100	−11.23

### Microscopy

**Fig. 2:**
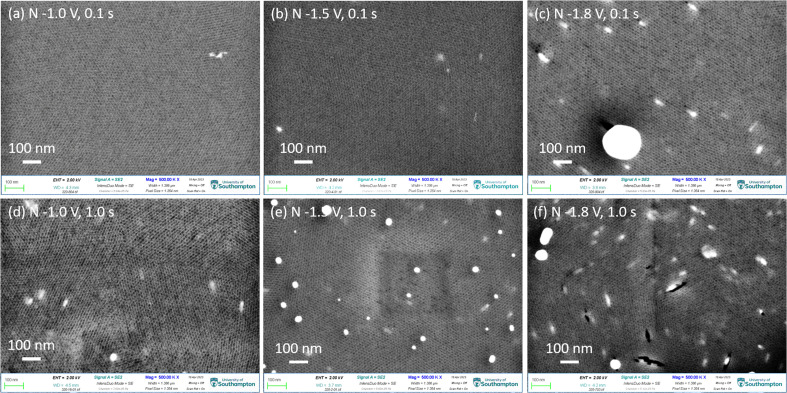
(a–f) Top-view SEM images of gold electrodeposited in the mesoporous silica films with different nucleation potentials and times, followed by growth at −0.1 V for 1.0 s for 100 cycles. The nucleation potentions and time for each sample were labelled in the images. “N” in each image represents the nucleation conditions.

Fig. 2 shows the top-view SEM images of gold electrodeposited in the mesoporous silica films with different nucleation potentials and times, followed by growth at −0.1 V for 1.0 s (pulse-on time) then at open circuit potential for 10.0 s (pulse-off time) for 100 repeated cycles. The highly ordered mesoporous silica films with pore diameters of 6–10 nm are observed and the thickness of films are around 150 nm from our previous study.^18^ The light irregular features are from the gold nanoparticles still in the thin films. It indicates that gold nucleated at the surface of the TiN substrate and then grew through the mesoporous silica film. More negative nucleation potential and longer nucleation time contribute to more gold nanoparticles reaching the surface of the thin film. When gold was nucleated at −1.0 V and −1.5 V for 0.1 s, there are few particles obtained on the surface as most gold particles are still in the film close to the TiN substrate. With a longer nucleation time of 1.0 s, samples nucleated at −1.0 V and −1.5 V show more gold features. When the nucleation potential was −1.8 V, there are a lot of gold nanoparticles observed both in the film and on the film. There are a few cracks containing gold nanoparticles, indicating that the deposited gold particles have larger sizes than the mesopores of the films, so the gold lacerated the film and grew through the crack until it got to the surface of the film, and then it kept growing on the surface. The top-view SEM images indicate that the nucleation potential and time can control the amount and growth of gold particles deposited in the film. Fig. S2† shows the EDX mapping and spectrum of sample 3 (nucleation at −1.5 V for 0.1 s), which demonstrate the particles on the surface are gold.

### X-ray diffraction

**Fig. 3:**
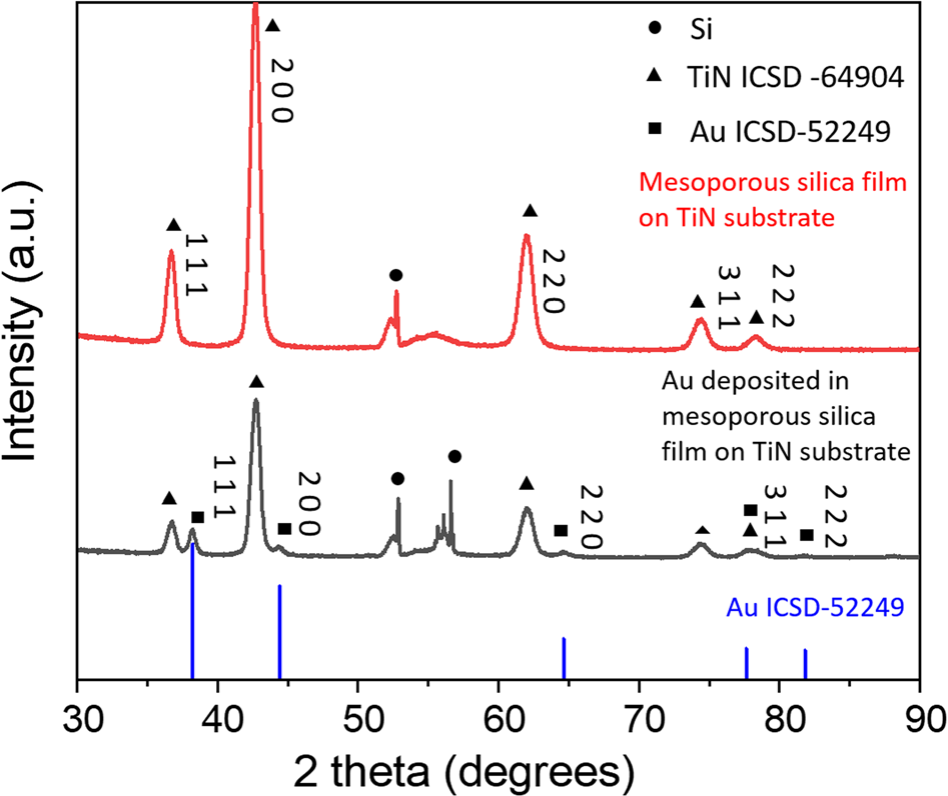
The XRD patterns of gold electrodeposited in the mesoporous silica film, the mesoporous silica film on TiN substrate and gold standard pattern ICSD-52249.

The gold electrodeposits in mesoporous silica films were characterised by XRD and they showed very similar XRD patterns. The XRD pattern of the typical sample 1 is shown in Fig. 3. The patterns of gold electrodeposited in the mesoporous silica films (black), the mesoporous silica film coated on the TiN substrate (red) and the standard pattern of gold *Fm*3*m* ICSD-52249 (blue) are shown. The peaks of TiN match well with *Fm*3*m* TiN ICSD-64904. The Miller indices of the key planes are labelled in the figure. The sharp peak at 52.9° comes from the silicon wafer of the substrate. After electrodeposition, *Fm*3*m* gold was observed. This confirms gold was prepared successfully through electrodeposition.

### GISAXS

**Fig. 4:**
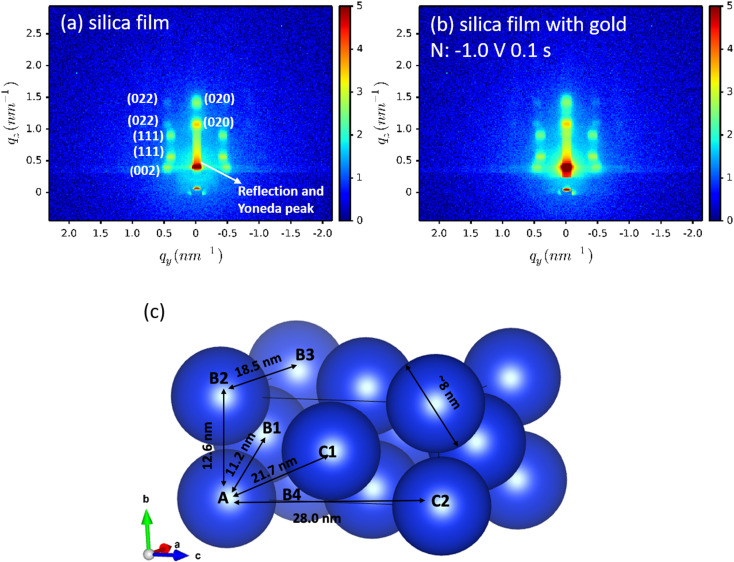
GISAXS patterns of the (a) mesoporous silica films before electrodeposition (silica) and (b) after gold electrodeposition (silica with gold). The incident angle was 0.3° and the samples were scanned for 20 min. The Bragg diffraction peaks are labelled in (a). “N” in each image represents the nucleation conditions. (c) Orthorhombic symmetry with space group *Fmmm* and the [0 1 0] axis perpendicular to the substrate (Substrate treated on a plane at the bottom of the diagram). The blue spheres represent mesopores. The distances between the neighbour pores are labelled.

For each sample, GISAXS was conducted on: (1) the mesoporous silica films; (2) the gold-containing film after electrodeposition; (3) the gold nanostructures after etching the films away by HF. The GISAXS patterns of the mesoporous silica films before (silica) and after gold electrodeposition (silica with gold) of a typical sample are shown in Fig. 4(a) and (b). All GISAXS patterns of the six samples in Table 1 are displayed in Fig. S3.† All the samples were scanned for 20 min with X-ray incident angle of 0.3°. Scattering angle can be converted to scattering vector using 
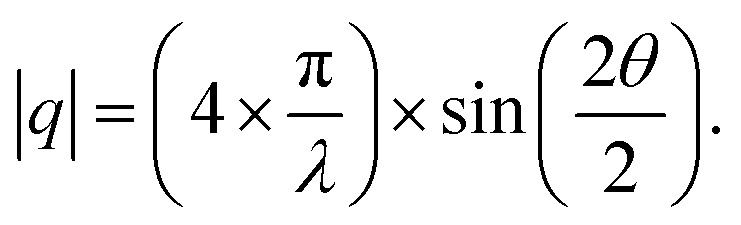
. Therefore, the specular reflection peak is at 0.6°, or 0.428 nm^−1^. The Yoneda peak is at 0.539°, corresponding to 0.384 nm^−1^.^44^ The array of intense spots in GISAXS is assigned to a well-ordered orthorhombic symmetry with space group *Fmmm* with the [0 1 0] axis perpendicular to the substrate.^18,45^ The *Fmmm* mesostructure consists of a face-centred cubic array of spherical mesopores with an orthorhombic distortion. This structure has previously been attributed to formation of a cage-like *Im*3*m* structure with [1 1 0] normal to the substrate that then contracts along this direction during the calcination process.^46^ A sketch of the *Fmmm* unit cell with the [0 1 0] axis perpendicular to the substrate is displayed in Fig. 4(c). The reflections in the obtained GISAXS patterns are doubled up, the lower *q*_z_ reflections are transmitted Bragg diffraction and the higher *q*_z_ reflections are specular reflections. The GIXSGUI software was used to predict the diffraction peaks and compare the calculated peaks directly with the experimental data.^47^ The GIXSGUI indexing images are shown in Fig. S4.† The cell parameters were measured as *a* = 18.5 nm, *b* = 12.6 nm and *c* = 28.0 nm. The indices of each pair of diffraction peaks are labelled in Fig. 4(a). For all samples in Fig. S3,† the pattern of Bragg peaks doesn't change significantly after gold electrodeposition, indicating that the *Fmmm* silica mesostructure still exists and the three-dimensional pore channels were not destroyed by the deposited gold. However, the small changes in peak positions and intensities can be caused by the deposited gold. Notably there are small variations in the GISAXS patterns of the mesoporous silica films even before electrodeposition, because the mesostructure is sensitive even to very small changes in experimental conditions such as humidity, temperature, heating rate *etc*. For each sample, the GISAXS patterns are obtained before and after gold electrodeposition to ensure the small changes in peak positions and intensities are from the deposition.

**Fig. 5:**
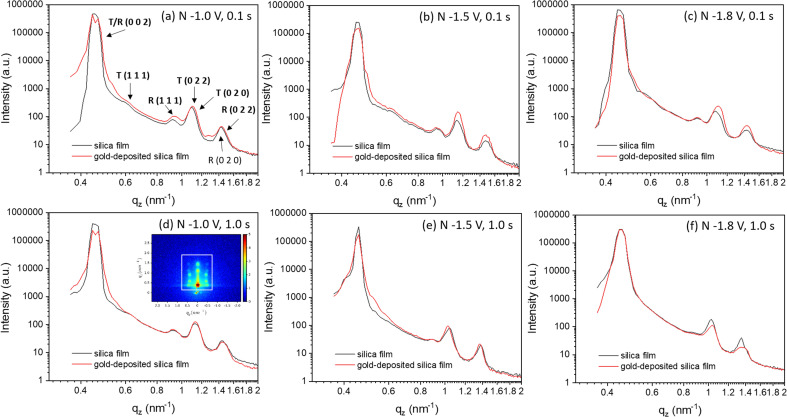
(a–f) Vertical-cut of GISAXS patterns of mesoporous silica film before and after gold electrodeposition from Fig. S2.† The vertical line cut area in GISAXS is shown in (d) by a white square. The peaks are labelled in (a). “*T*” represents transmission and “*R*” represents reflection. The nucleation potentials and time were labelled in each images. “N” in each image represents the nucleation conditions.

Vertical (Fig. 5 and S5†) and horizontal (Fig. S6†) cuts were extracted from the GISAXS patterns in Fig. S3† using the DPDAK software package.^48^ Vertical scattering profiles give information about the structure along the vertical direction while horizontal-cuts do so for the horizontal direction. The scattering of X-rays arises from the electrons in the shells, thus it generally increases with the atomic number. At 8 keV, the values of the dispersive part *δ* of the refractive index (*δ* = 1 − *n* + *iβ*, *n*: complex refractive index, *β*: absorbative part) are 4.7730 × 10^−5^ for gold and 7.21221295 × 10^−6^ for silica.^49^ Therefore, gold scatters X-rays more strongly than silica. Before electrodeposition, the patterns reflect the contrast between the silica walls of the mesoporous structure and air. When gold starts to electrodeposit into the mesopores and fills them over a wide area, it could be expected that the intensity of the GISAXS peaks would be stronger since gold is denser and silica and air is less dense. In Fig. 5 and S5,† after gold electrodeposition, the intensities increase in most samples as expected, indicating there are widespread gold deposits with structure in the vertical direction from each set of applied electrodeposition conditions. The intensity of the sample nuclei at −1.8 V for 1.0 s decreased after electrodeposition, which could be caused by an initial lower contrast as a small number of pores filled with gold while most remained empty. The consistent peak positions demonstrate that the gold is structured with the same periodicity as observed in the original silica structure.

The cutting area is shown in the white square area in Fig. 5(d). In Fig. 5, the peak with highest intensity at around 0.4 nm^−1^ corresponds to the specular reflection (0.428 nm^−1^) and Yoneda peak (0.384 nm^−1^). The six peaks between 0.4 and 1.8 nm^−1^ are from the 0 0 2, 1 1 1, 0 2 0 and 0 2 2 peaks of the mesoporous structure. The 0 0 2 peak is too close to the strong specular reflection for its position to be accurately measured from the pattern in Fig. 5. The 0 2 0 peaks have high intensity and they are close to the 0 2 2 peaks, so the 0 2 2 peaks can only be observed well by subtracting the 0 2 0 peaks. Therefore, Fig. S5† subtracts the vertical-area along specular reflection and Yoneda peak to obtain a stronger signal from peaks 0 2 2, 0 0 2 and 1 1 1. The positions of peaks 0 0 2, 1 1 1 and 0 2 2 are obtained from Fig. S5† and those of 0 2 0 are from Fig. 5. The positions of these peaks and the position changes of the observed peaks before and after gold electrodeposition are listed in Table S1.†

For all the samples, the position of the 0 0 2 peaks does not change after depositing gold in the film, showing that the unit cell size does not change along the *c* axis. As the [0 1 0] axis is perpendicular to the substrate, the *c*-axis represents one of the directions parallel to the substrate. Fig. 4(c) shows a sketch of the *Fmmm* unit cell, where the blue spheres are the pores. The lattice parameters *a* = 18.5 nm, *b* = 12.6 nm and *c* = 28.0 nm. In the ab plane the pores are significantly closer to each other, so connectivity from sphere “A” to “B1”, “B2” or “B4” will be through an open gap or a thin pore wall, whereas connectivity to sphere “C1” or “C2” would involve a much thicker pore wall. While mesoporous silica structures often have microporosity in the pore walls, this means connectivity along the *c* axis is much harder to achieve. Therefore, gold nanoparticles tend to grow in the ab plane, resulting in no shifts in the 0 0 2 reflection position. Growth along the *b* axis may also be favoured by the diffusion process through the thickness of the silica film, although it should be noted that the pulsed deposition process was designed to minimise this effect.

The positions of the peaks 1 1 1, 0 2 0 and 0 2 2 shift when gold is grown into the mesopores, suggesting the compression or swelling of the pore structure after gold electrodeposition. The *d*-spacings can be calculated from the q values in Table S1† by 
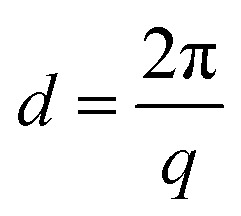
. The lattice parameters (*a*, *b* and *c*) can be then obtained by fitting the 0 2 0, 0 0 2 and 1 1 1 d-spacing data in 
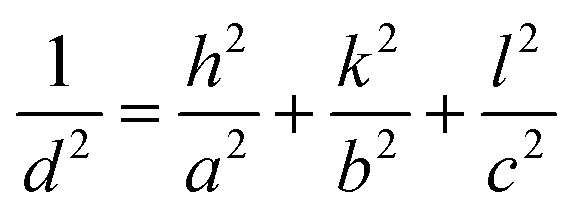
. The lattice parameters of each sample before and after gold electrodeposition are listed in Table 2. It is noteworthy that where changes are seen the largest changes are in the *a* axis direction, parallel to the substrate, which may suggest that growth in one mesopore may aid nucleation at the substrate surface in a neighbouring mesopore along this direction where the pores are fairly close together.

**Table 2:** The lattice parameters of each sample before and after gold electrodeposition^a^

Sample no.	*a* (nm)			*b* (nm)			*c* (nm)		
	Before	After	Change	Before	After	Change	Before	After	Change
1	11.580	11.259	−0.321	8.880	8.800	−0.080	28.303	28.303	0
2	11.450	11.450	0	8.715	8.715	0	28.239	28.239	0
3	11.599	11.599	0	8.491	8.491	0	27.558	27.558	0
4	12.197	11.995	−0.202	9.054	9.139	+0.085	27.498	27.498	0
5	12.281	11.760	−0.521	8.800	8.800	0	28.303	28.303	0
6	12.039	12.039	0	9.322	9.322	0	28.239	28.239	0

a “Before” and “after” represents the silica film before and after gold electrodeposition; the parameter values *a*, *b* and *c* are calculated from the positions of the 1 1 1, 0 2 0 and 0 0 2 reflections, respectively; sample no. corresponds to that in Table 1.

### Removing the mesoporous silica to expose the gold

Removing the mesoporous silica films after electrodeposition is helpful to observe the morphology and arrangement of the deposited gold. Silica removal generally can be achieved with hydrofluoric acid (HF)^50,51^ or with sodium hydroxide/potassium hydroxide solutions.^52,53^ However, wet operations could damage the fragile nanostructure. Sodium hydroxide was used to remove the silica template. As an example, sample 3 (nucleation at −1.5 V for 0.1 s) was bathed into 2 mol L^−1^ sodium hydroxide aqueous solution for one hour. The top-view SEM images and EDX are displayed in Fig. S7.† There are no mesoporous silica films and gold particles observed like in Fig. 2(d) and S2.† A few irregular fragments were obtained on the surface, which could come from the damaged films. EDX then shows no gold to be present on the surface. Moreover, the oxygen content is three times lower than the sample before bathing in the sodium hydroxide solution which indicates the removal of silica film. However, the solution damaged the gold nanostructures when removing the silica film.

To avoid damage from exposing the gold deposits to liquid etchants, hydrofluoric acid vapour was used to remove the mesoporous silica. Previous publications have shown noble metals Pt and Au to have good resistance to hydrofluoric acid and thus the gold deposit remains after HF etching.^54^ During the vapour etching, the samples were supported above the mouth of the hydrofluoric acid bottle and the gold-deposited mesoporous silica film side faced the hydrofluoric acid vapour. All etched samples were exposed to hydrofluoric acid vapour for 20 minutes.

**Fig. 6:**
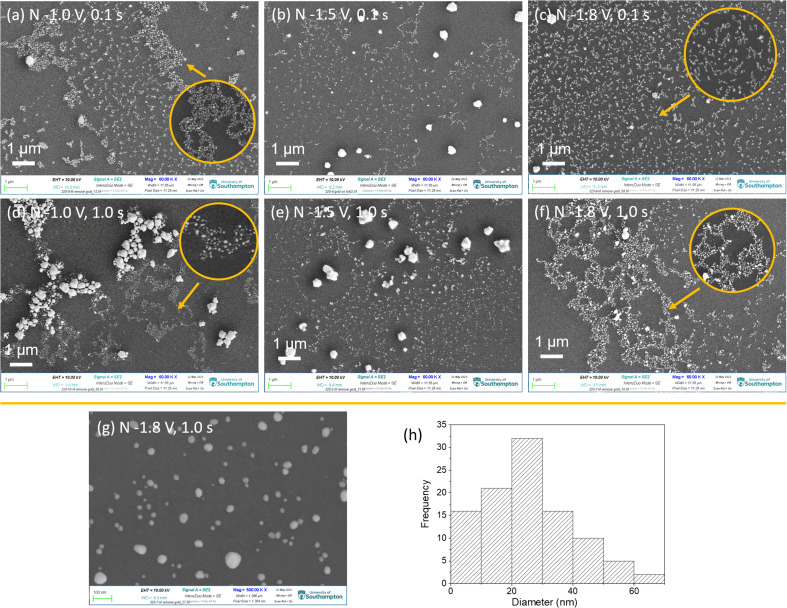
(a–f) Top-view SEM images of the samples of gold electrodeposited into mesoporous silica/TiN substrates after removing the silica using HF vapour. The magnification is 600 00×. The nucleation conditions are labelled in the images, “N” represents nucleation. (g) A top-view SEM image with magnification of 5 000 00× of sample nuclei at −1.8 V for 1.0 s, the particles size distribution of (g) is shown in (h).

After removing the film templates, the top-view SEM images of the above samples display interesting “fractal-like” features with different length of branches, as shown in Fig. 6(a–f). These branches consist of gold nanoparticles. As a typical example, the 500 000 magnification SEM image of sample nuclei at −1.8 V for 1.0 s was as shown in Fig. 6(g) and (h), shows the nanoparticles' size distribution in (g). The diameters of most nanoparticles are under 70 nm and most of them are between 10 and 30 nm, which are larger than the pore sizes (∼6 to 12 nm). Hence it appears that during growth the particles break through the walls of the mesoporous structure.

With change of nucleation potential and time, gold nanoparticles display different arrangements. There are three major arrangements: (1) individual and denser nanoparticles; (2) fractal morphology with the length of branch varying with the deposition conditions; (3) the micro-scale gold aggregates, which would not be expected to be observed with GISAXS. These arrangements can exist in one sample. When applying the least negative potential −1.0 V for the shortest time 0.1 s, individual nanoparticles and fractal morphology were both observed. With longer time nucleation (1.0 s) at −1.0 V, the deposited gold nanoparticles interlaced over a wide area to form a fractal morphology with long chains. Also, a large number of micro-size gold aggregates were formed. When applying the most negative potential of −1.8 V for 0.1 s, gold with even short-chain fractal structures were obtained. However when nucleation time was longer (−1.8 V for 1.0 s), long-chain fractal structures and a few micro-size particles were obtained. With nucleation potential of −1.5 V, most gold showed individual particles or very short chain fractal structures and micro size particles.

As a comparison, gold particles were electrodeposited on the bare TiN substrates under the same deposition conditions as in Table 1. Fig. S8† shows the top-view SEM images of them. Rather than fractal morphology, gold nanoparticles obtained from template-free electrodeposition show individual particles dispersed on the substrates at various densities. Fig. S9† displays the particle diameter distributions from the SEM images in Fig. S8.† Most of the deposited gold particles are above 50 nm in diameter, which are generally larger than the gold deposited with silica film templates. When applying potentials for a short nucleation time (0.1 s), the obtained gold particle sizes are barely under 50 nm. When nucleation time is longer (1.0 s), more small particles appear, however, there is still a large number of big particles with diameter larger than 50 nm. It indicates that the silica film templates contribute to the formation of smaller size gold deposits. Our previous study has investigated the spatial correlations within the deposited particles on bare substrates by using GISAXS.^41^

Small-angle scattering (SAXS) curves can be divided into the Guinier region and the Porod region. The Guiner–Porod empirical model demonstrates how the scattering intensity is defined by the radius of gyration and the Porod exponent, as shown in eqn (1) and (2). The Guinier region is at low values of the scattering vector *q*, from which the radius of gyration can be calculated by eqn (1) regardless of the particle shape (Guinier law). At larger *q* values, Porod's law describes the asymptote of the scattering intensity *I*(*q*) with *q* by eqn (2). This region generally gives information about the specific surface and fractal dimensions of the investigated sample.^55^1
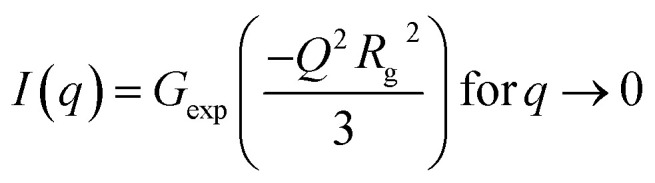
2
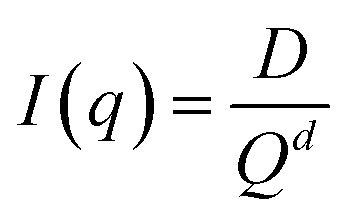
where *q* is the scattering vector, *I*(*q*) is the scattered intensity, *R*_g_ is the radius of gyration, *d* is the Porod exponent, *G* and *D* are the Guinier and Porod scale factors.^56,57^

**Fig. 7:**
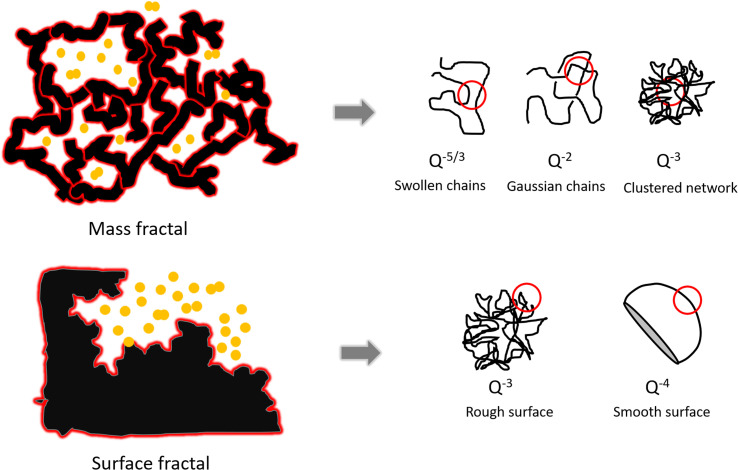
The schematic of mass fractal and surface fractal and Porod law for different shape of objects.^60^

“Fractal surfaces” are widely used to describe polymer structures. A mass fractal is a structure with branching and crosslinking to form a 3D network. In a surface fractal, only the surface is fractal. Fig. 7 displays assortment of fractal Porod exponents. When Porod exponents *d* < 3, particles are “mass fractals”, when 3 < *d* < 4, particles are “surface fractals”. Moreover, *d* = 4 indicates that particles have smooth surfaces, while *d* = 3 represents the very rough surfaces. *d* = 5/3 shows the scattering from ‘fully swollen’ chains. *d* = 2 indicates the scattering either from Gaussian polymer chains or from a two-dimensional structure.^58^ An exponent *d* = 1 is obtained for scattering from rigid rods (or thin cylinders).^59^ The model is used to describe SAXS data as an approximation. It has not previously been used to describe metal particles in GISAXS.

**Fig. 8:**
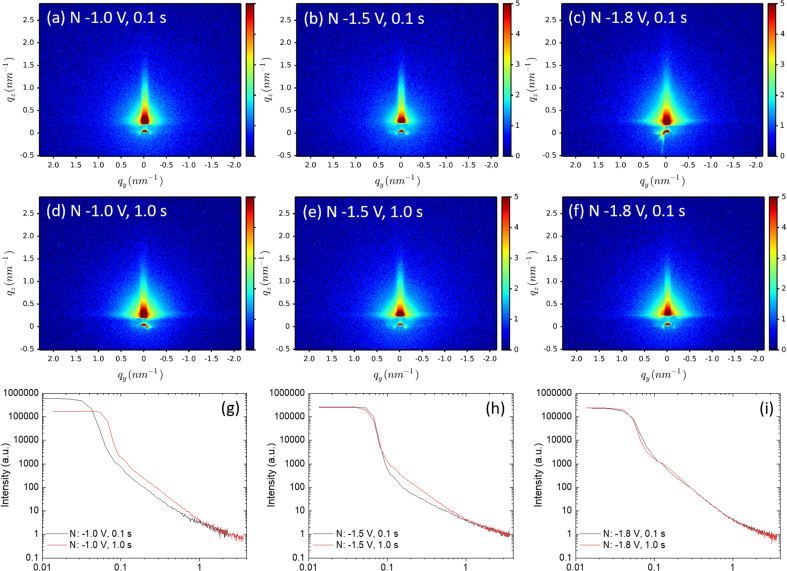
(a–f) 2D-GISAXS patterns of the gold-electrodeposition samples after removing the silica film. The incident angle is 0.2° and the sample is scanned for 20 minutes. The horizontal-cut curves (g)–(i) were extracted from GISAXS patterns using DPDAK. The samples which share the same nucleation time are shown in the same graph. The fitting graphs and results are shown in Fig. S10.†

The 2D-GISAXS patterns of gold-electrodeposition samples after removing the silica film are shown in Fig. 8(a–f), as well as the horizontal-cut curves extracted from the obtained GISAXS patterns (g–i) after removing the silica film, the *Fmmm* mesostructure is no longer observed. The GISAXS patterns show that the gold particles distribute on the substrates without a well-ordered structure, presumably because the gold structures collapse onto the surface during the treatment with HF. As the fractal morphologies were observed by SEM, Porod's law is therefore used to investigate the fractal dimensions of these samples. The SAXS data are obtained from the horizontal cuts of the GISAXS patterns in Fig. 8, the cuts are taken parallel to the sample surface that they can be treated as conventional SAXS patterns.^57^

Porod exponents d were obtained from fitting the Porod region of the horizontal-cut curves by using eqn (2). The Porod fitting graphs and fitting results of each sample are shown in Fig. S10.† The *R*^2^ values were controlled to be higher than 0.99. The *d* values are displayed in Table 3. When *d* is between 2 and 3, smaller d gives a more even surface and larger *d* gives a rougher surface.^61^ The *d* values of all nine samples are smaller than 3, indicating they are all mass fractals. The *d* value of sample nuclei at –1.8 V for 1.0 s is 2.97, very close to 3, it suggests that the sample has rough and high folded surface like a clustered network, which is consistent with the SEM image in Fig. 6(f). The *d*-values of sample nuclei at −1.5 V for 0.1 s are below 2, which represents simple chains without overlapping, can be seen in the SEM image in Fig. 6(b): the gold particles display very short chains.

**Table 3:** The value of the Porod exponent *d* fitting from the Porod area of Fig. S10

Time s	Potential V		
	−1.0 V	−1.5 V	−1.8 V
0.1 s	2.17 ± 0.01	1.92 ± 0.03	2.50 ± 0.02
1.0 s	2.52 ± 0.01	2.40 ± 0.02	2.97 ± 0.01

Table 3 shows the variation in the GISAXS patterns as the nucleation time increases from 0.1 to 1.0 s. The *d*-values increase, indicating longer nucleation time gives a rougher mass fractal surface. When the nucleation potential was fixed at −1.0 V, and the nucleation time increased from 0.1 to 1.0 s, the particle arrangements changed from bulk particles with short chains to branched structures with long chains. Similarly, when applying nucleation potential −1.8 V, short chains were obtained with 0.1 s nucleation time and long chains, with surface fractal-like structures obtained with 1.0 s. From the obtained *d* values and SEM images, generally with the increase of nucleation time, fractal gold nanoparticles with longer branches tend to be obtained.

## Conclusions

Fractal-like gold nanoparticles were obtained by templated electrodeposition with 3D mesoporous silica films as the templates. Mesoporous silica films are insulating so the deposited gold formed nuclei on the conductive substrates and then grew along the mesoporous channels. Nucleation potential and time were controlled. The spatial arrangement of gold nanoparticles from templated electrodeposition were investigated with GISAXS and SEM. The deposited gold shows significantly different morphologies compared with deposition on flat surfaces, indicating the templates play an important role in controlling the growth of the often interlinked gold particles during the electrodeposition. Moreover, the gold particles deposited on bare TiN substrates are larger than those deposited with silica film templates. It indicates that the silica film templates can limit the sizes of the deposited gold nanoparticles. Comparing the GISAXS patterns before and after electrodeposition, gold nanoparticle chains grew preferentially in the ab planes as observed *via* changes in the 1 1 1, 0 2 0 and 0 2 2 peak positions. This is because the mesopores in the ab planes are closer together than those along *c*, or even directly connected in some cases. The intensities of peaks in the horizontal-cuts did not change significantly, indicating that there were no uniform wide-area gold nanowires electrodeposited in the mesopores. The Guiner–Porod empirical model was used to describe the fractal morphology of gold after removing the silica film templates. The Porod exponent can be obtained by fitting the GISAXS patterns of gold. The gold arrangements of all samples can be described by mass fractals with different roughnesses. Longer nucleation time generally contributes to formation of rougher surfaces of gold fractal structures with longer branches.

## Conflicts of interest

The authors declare no conflicts of interest.

## Supplementary Material

RA-013-D3RA06588J-s001
