# Supplementary material for: Fractal-like gold nanonetworks formed by templated electrodeposition through 3D-mesoporous silica films
Source: RSC Adv. 2023 Nov 6;13(46):32660–71. doi: 10.1039/d3ra06588j (PMC10626528; doi:10.1039/d3ra06588j)
Supplement: RA-013-D3RA06588J-s001 [file RA-013-D3RA06588J-s001.pdf]

Electronic Supplementary Material (ESI) for RSC Advances.
This journal is © The Royal Society of Chemistry 2023

Supplementary Information: **Fractal-like gold nanonetworks formed by templated electrodeposition through 3D-mesoporous silica films**

Li Shao,*[a] Gilles E. Moehl,[a] Ruomeng Huang[b] and Andrew L. Hector*[a]

*Corresponding authors

[a.] School of Chemistry, University of Southampton, Highfield, Southampton SO17 1BJ, UK.

Email : A.L.Hector@soton.ac.uk; Li.Shao@soton.ac.uk

[b.] School of Electronics and Computer Science, University of Southampton, Southampton, SO17 1BJ, UK.

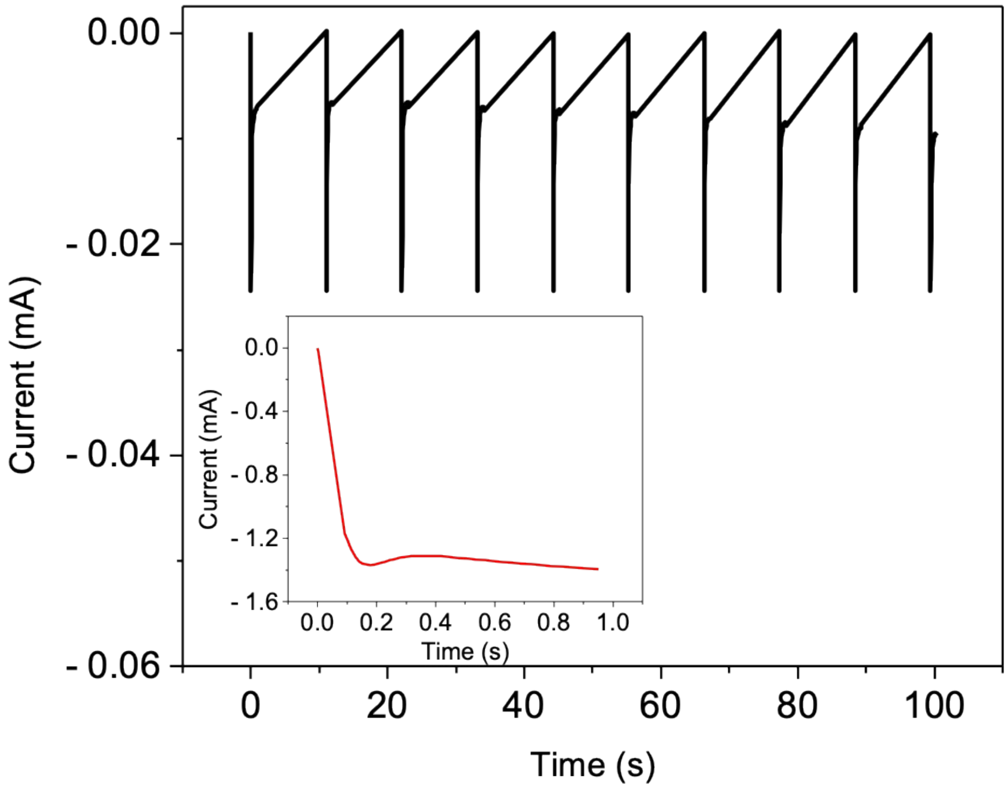

Figure S1. Current transients during pulsed potential deposition in 0.5 mM K[$AuCl_4$] and 100 mM KCl aqueous solution: nucleation at −1.8 V for 1.0 s (current transient shown in inset), followed by growth at −0.1 V for 1 s for 100 cycles. First 10 cycles are shown here.

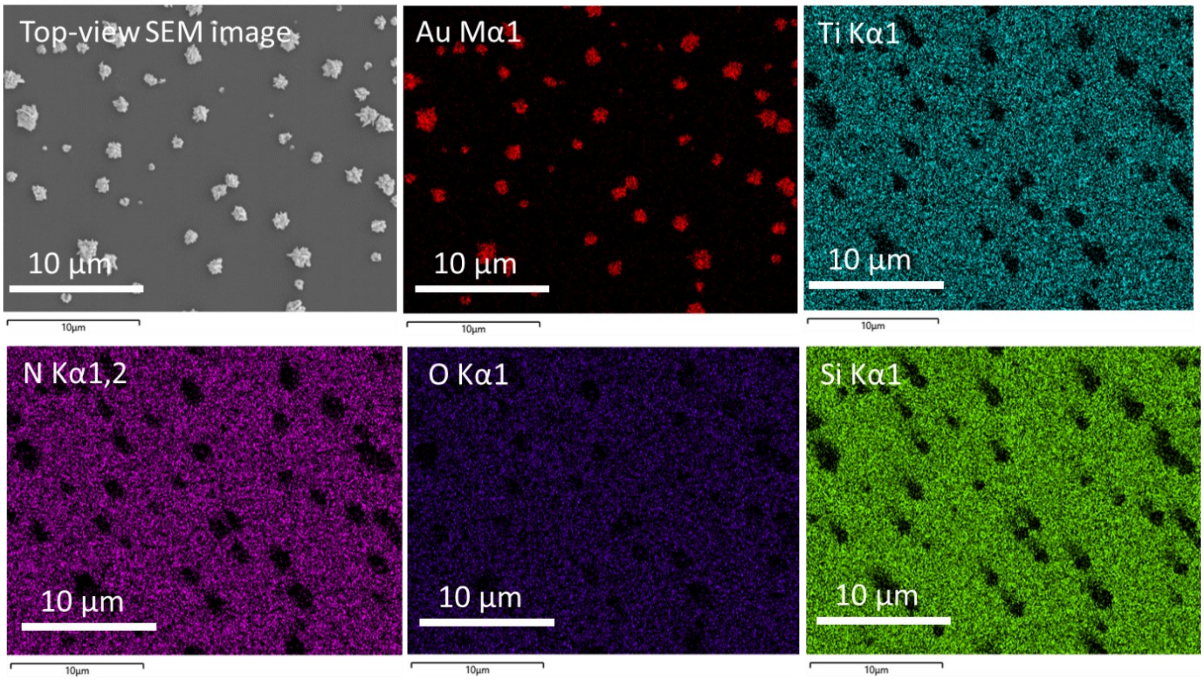

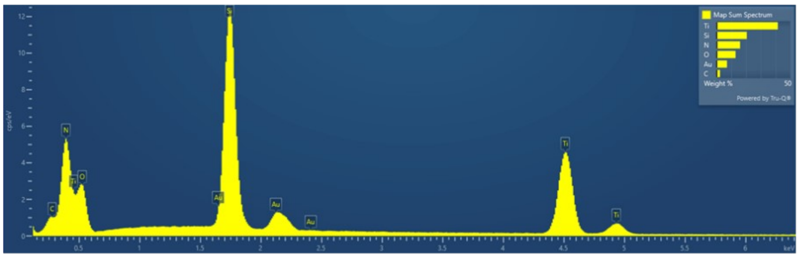

Element	wt%
Au	7.01±0.14
Si	20.42±0.11
O	12.86±0.16
Ti	41.51±0.20
N	15.90±0.22
C	2.30±0.15

Figure S2 Energy-dispersive X-ray (EDX) elemental mapping analysis. EDX mapping of elements Au, Ti, N, O and Si and EDX spectrum of sample 3.

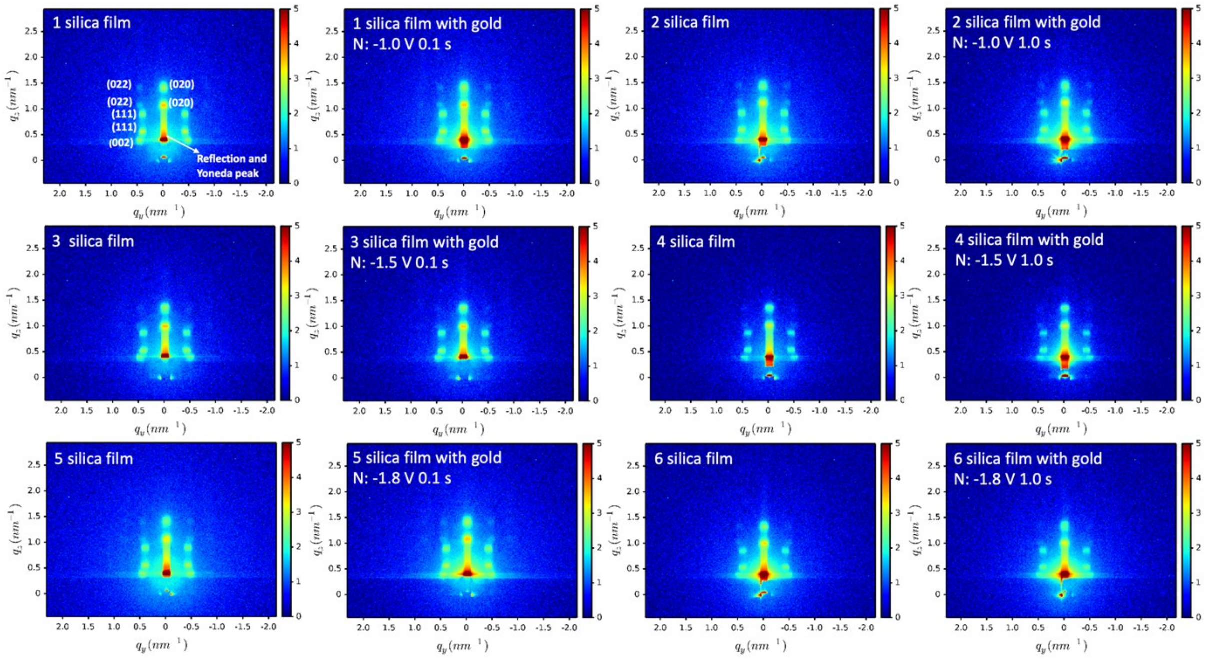

Figure S3. GISAXS patterns of the mesoporous silica films before (silica) and after gold electrodeposition (silica with gold). The electrodeposition conditions for each sample are shown in Table 1. The incident angle was 0.3° and the samples were scanned for 20 min. The Bragg diffraction peaks are labelled in 1 silica film. “N” represents the nucleation conditions.

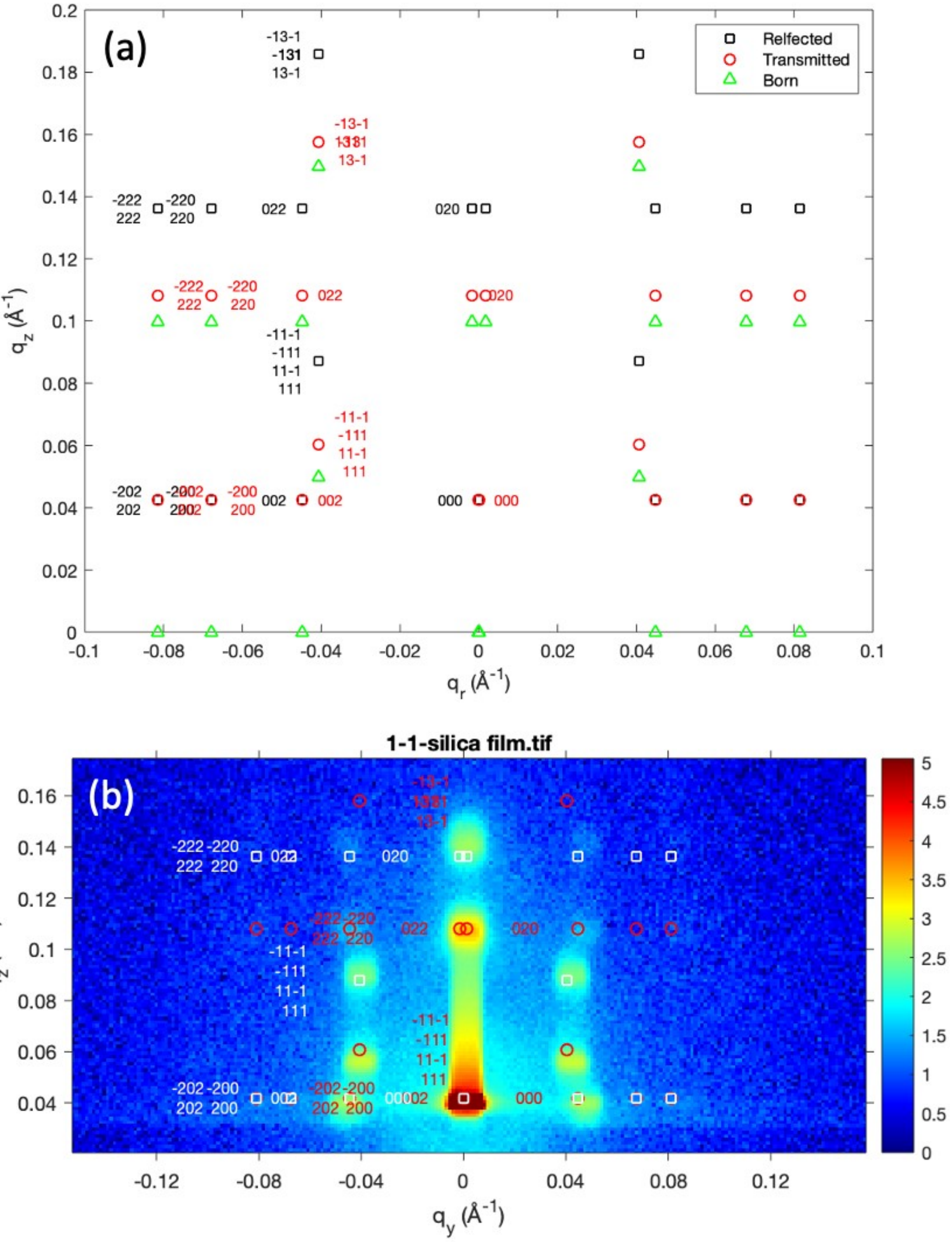

Figure S4. (a) The simulated Bragg peaks calculated by GIXSGUI software. Simulated patterns: *Fmmm* with (0 1 0) plane vertical to the substrate. Simulated lattice parameters values: a = 18.5 nm, b = 12.6 nm c = 28.0 nm; (b) The experimental Bragg peaks covered by the simulated Bragg peaks in (a). The red circles and white squares represent the transmitted and reflected Bragg peaks, respectively.

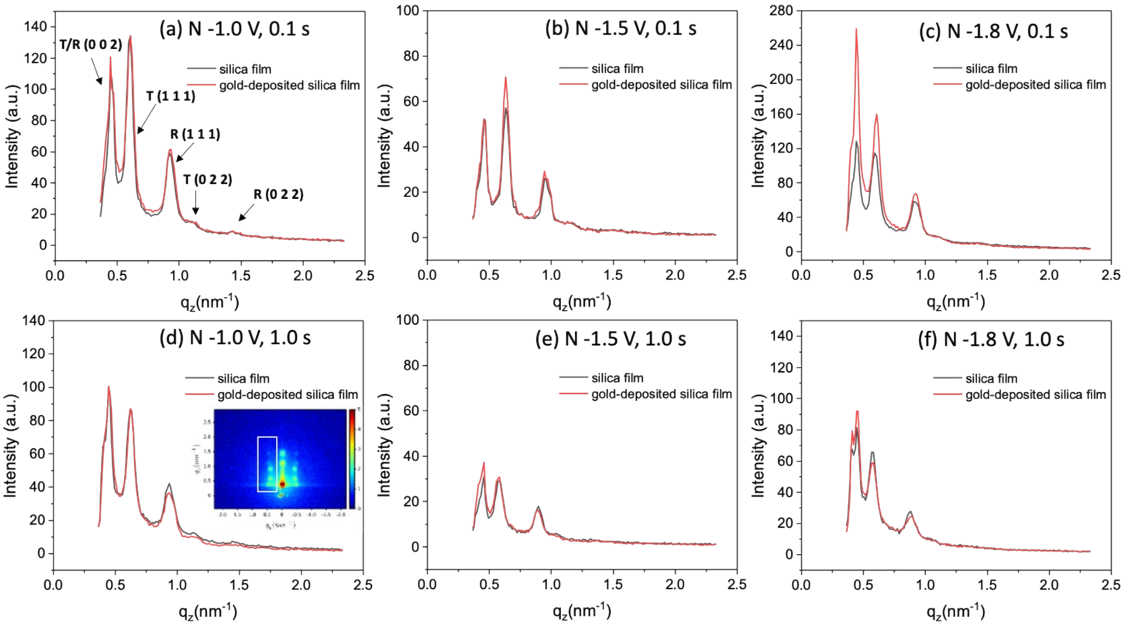

Figure S5. Vertical-cut of GISAXS patterns of mesoporous silica film before and after gold electrodeposition from Figure S2. The vertical line cut area in GISAXS is shown in (d) by a white square. The peaks are labelled in (a). “T” represents transmission and “R” represents reflection.

Table S1 The peak positions from vertical-cut GISAXS patterns from Figure 4 and Figure S4.

Sample No.	Transmitted /reflected 002 (nm^{-1})	Transmitted 111 (nm^{-1})	Reflected 111 (nm^{-1})	Transmitted 022 (nm^{-1})	Reflected 022 (nm^{-1})	Transmitted 020 (nm^{-1})	Reflected 020 (nm^{-1})
1 before	0.444	0.606	0.919	1.080	1.415	1.093	1.428
1 after	0.444	0.606	0.933	1.093	1.428	1.106	1.442
Peak move	0	0	+0.013	+0.013	+0.013	+0.014	+0.013
2 before	0.445	0.619	0.933	1.120	1.442	1.132	1.442
2 after	0.445	0.619	0.933	1.120	1.442	1.145	1.442
Peak move	0	0	0	0	0	+0.013	0
3 before	0.456	0.631	0.945	1.158	1.361	1.132	1.480
3 after	0.456	0.631	0.945	1.158	1.347	1.145	1.480
Peak move	0	0	0	0	-0.013	+0.013	0
4 before	0.457	0.581	0.894	1.040	-	1.040	1.388
4 after	0.457	0.581	0.894	1.026	-	1.026	1.375
Peak move	0	0	0	- 0.014	0	- 0.013	- 0.013
5 before	0.444	0.593	0.906	-	-	1.079	1.428
5 after	0.444	0.606	0.919	-	-	1.106	1.428
Peak move	0	+0.013	+0.013	-	-	+ 0.027	0
6 before	0.445	0.581	0.881	-	-	1.027	1.348
6 after	0.445	0.568	0.881	-	-	1.040	1.348
Peak move	0	+0.013	0	-	-	+ 0.013	0

“before” and “after” represents the silica film before and after gold electrodeposition; The positions of peaks 0 0 2, 1 1 1 and 0 2 2 are obtained from Figure S4 and that of 0 2 0 are from Figure 4; Sample No. corresponds to that in Table 1.

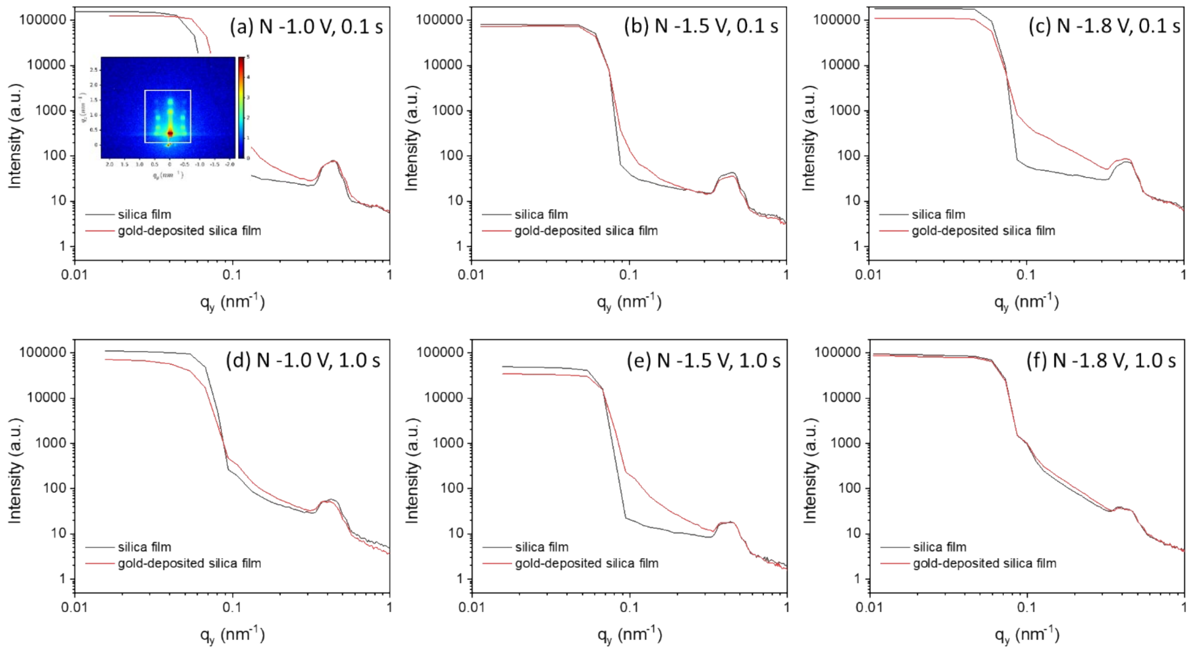

Figure S6. Horizonal-cut of GISAXS patterns of mesoporous silica film before and after gold electrodeposition from Figure S2. The horizonal-cut area in GISAXS is shown in the inset of (a) by a white square.

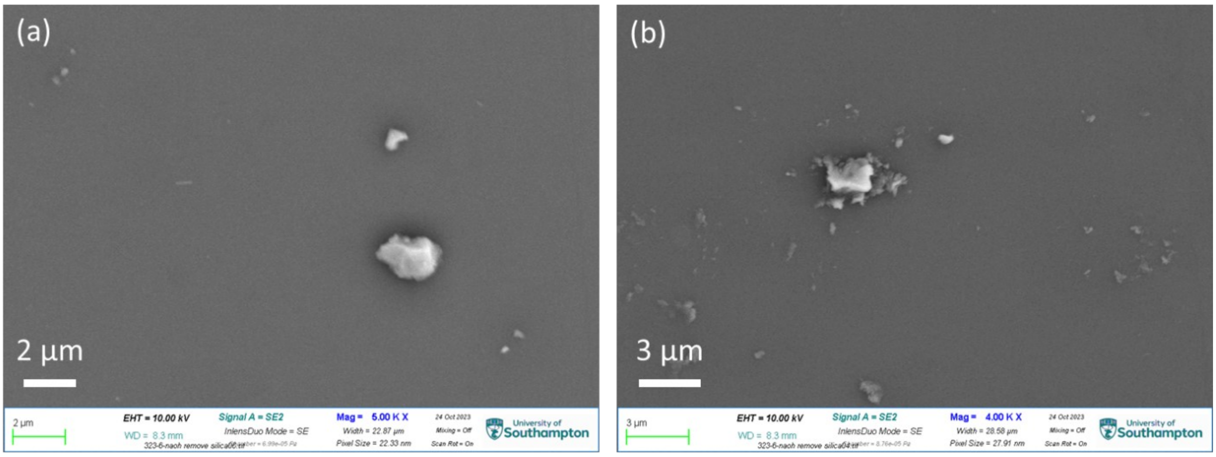

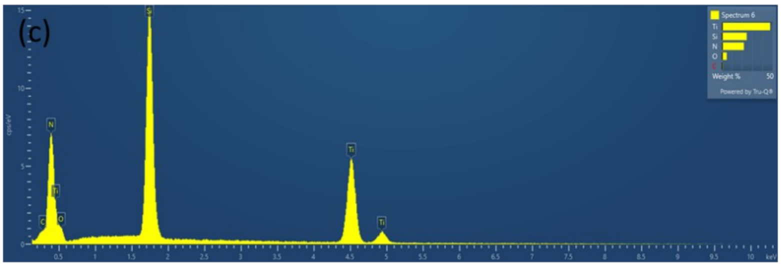

(d)

Element	wt%
Si	24.59±0.27
O	4.82±0.35
Ti	47.67±0.47
N	21.85±0.49
C	1.07±0.38

Figure S7. (a,b) Top-view SEM images of sample 3 after bathing in 2 mol/L sodium hydroxide solution for one hour; (c,d) EDX spectrum and the element content table of SEM image (a).

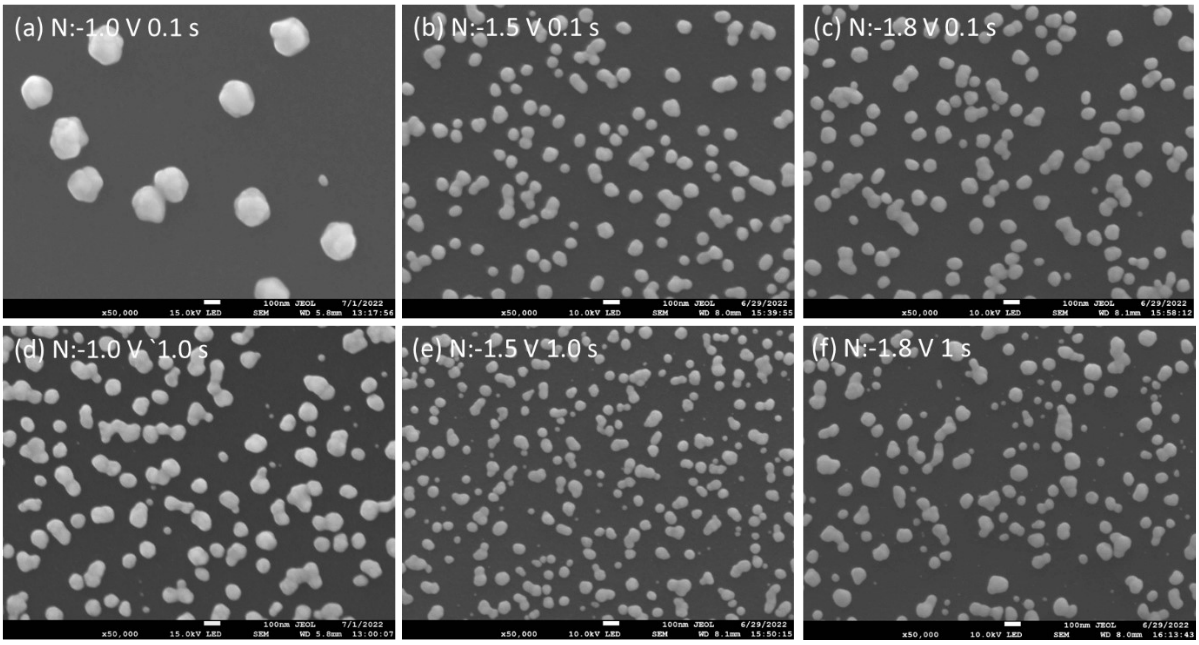

Figure S8. Top-view SEM images of gold electrodeposited on bare TiN substrates with a magnification of 50, 000 x. Gold electrodeposition was conducted by applying nucleation potentials at -1.0 V, -1.5 V and -1.8 V for 0.1 s and 1.0 s respectively, followed by growth at -0.1 V for 1 s for 100 cycles. The nucleation conditions are labelled in the images, “N” represents nucleation.

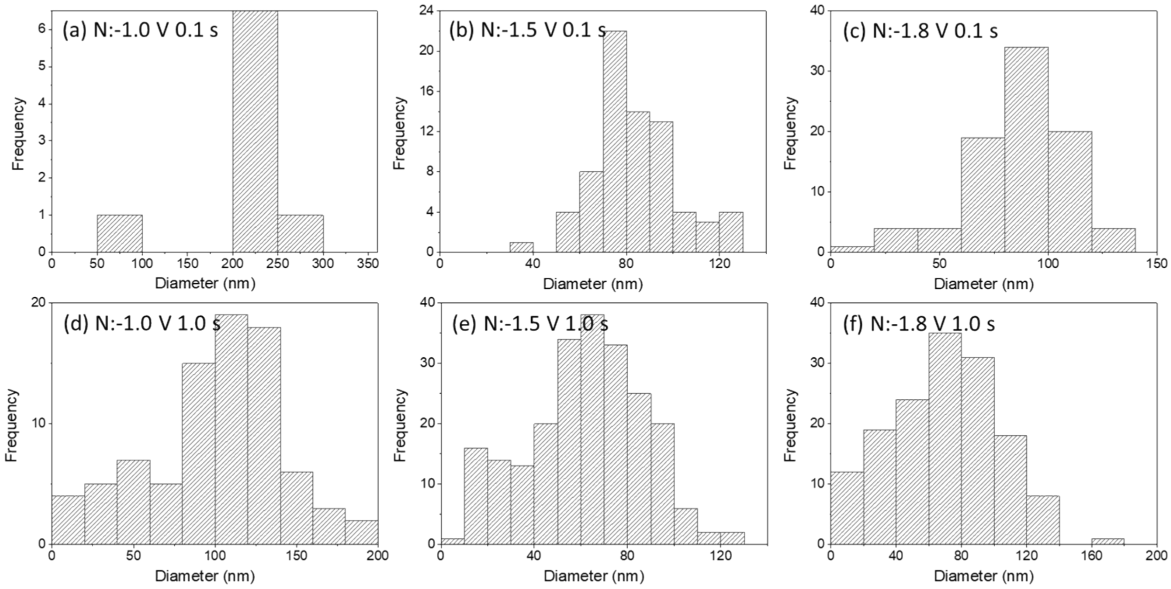

Figure S9. The particle diameter distribution from the SEM images in Figure S8.

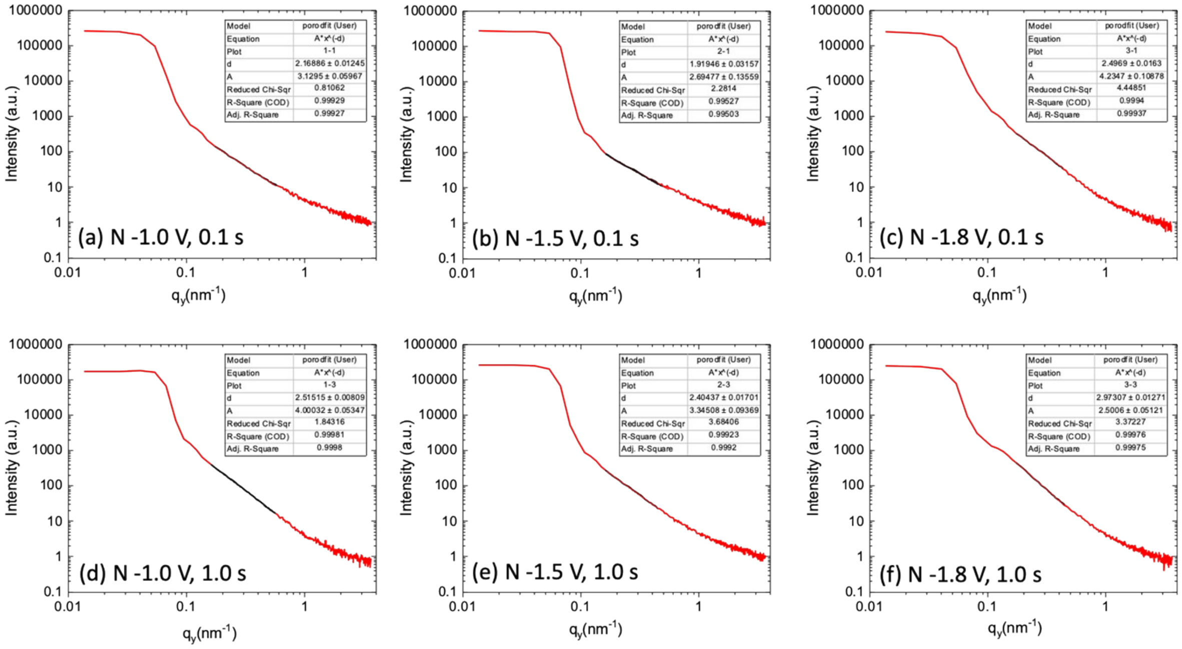

Figure S10. The horizonal-cut curves extracted from the GISAXS patterns in Figure 7 using DPDAK. The fitting areas are shown by a black line. The fitting results are shown in each image.
